# *Panax quinquefolius* Polysaccharides Ameliorate Antibiotic-Associated Diarrhoea Induced by Lincomycin Hydrochloride in Rats via the MAPK Signaling Pathways

**DOI:** 10.1155/2022/4126273

**Published:** 2022-03-19

**Authors:** Duo-duo Ren, Shan-shan Li, Hong-mei Lin, Yun-shi Xia, Zhi-man Li, Pan-pan Bo, Rui Mu, Li-juan Zhao, Yin-shi Sun

**Affiliations:** ^1^College of Chinese Medicinal Materials, Jilin Agricultural University, Changchun 130118, China; ^2^Institute of Special Wild Economic Animals and Plants, Chinese Academy of Agricultural Sciences, Changchun 130112, China; ^3^Institute of Biological and Pharmaceutical Engineering, Jilin Agricultural Science and Technology University, Jilin 132101, China

## Abstract

American ginseng (*Panax quinquefolius* L.) is an herbal medicine with polysaccharides as its important active ingredient. The purpose of this research was to identify the effects of the polysaccharides of *P. quinquefolius* (WQP) on rats with antibiotic-associated diarrhoea (AAD) induced by lincomycin hydrochloride. WQP was primarily composed of galacturonic acid, glucose, galactose, and arabinose. The yield, total sugar content, uronic acid content, and protein content were 6.71%, 85.2%, 31.9%, and 2.1%, respectively. WQP reduced the infiltration of inflammatory cells into the ileum and colon, reduced the IL-1*β*, IL-6, IL-17A, and TNF-*α* levels, increased the levels of IL-4 and IL-10 in colon tissues, improved the production of acetate and propionate, regulated the gut microbiota diversity and composition, improved the relative richness of *Lactobacillus* and *Bacteroides*, and reduced the relative richness of *Blautia* and *Coprococcus*. The results indicated that WQP can enhance the recovery of the intestinal structure in rats, reduce inflammatory cytokine levels, improve short-chain fatty acid (SCFA) levels, promote recovery of the gut microbiota and intestinal mucosal barrier, and alleviate antibiotic-related side effects such as diarrhoea and microbiota dysbiosis caused by lincomycin hydrochloride. We found that WQP can protect the intestinal barrier by increasing Occludin and Claudin-1 expression. In addition, WQP inhibited the MAPK inflammatory signaling pathway to improve the inflammatory status. This study provides a foundation for the treatment of natural polysaccharides to reduce antibiotic-related side effects.

## 1. Introduction

Antibiotic-associated diarrhoea (AAD) is a common negative side effect of antibiotic treatment and involves gut microbiota destruction, reduced concentrations of intestinal short-chain fatty acids (SCFAs), accumulation of intestinal carbohydrates and colonic cholic acid, changes in water absorption, and ultimately diarrhoea [1]. Gut microbiota dysbiosis is a major feature of AAD [2]. As a new “organ,” the gut microbiota is largely involved in nutrition, immunity, metabolism, digestion, and other physiological functions and is critical for maintaining homeostasis of the body [3]. In general, the gut microbiota and the host maintain a relatively balanced relationship. Once this balance is disrupted, gut microbiota disorder can lead to the development of disease [4]. The gut microbiota is linked to many diseases, including inflammatory bowel disease [5], diabetes [6], cirrhosis [7], hypertension [8], and obesity [9]. Maintaining healthy gut microecology is very important to the health of the host.

Herbal medicine can serve as valuable therapeutics that are vital in the medical applications of Asian countries [10]. American ginseng (*Panax quinquefolius* L.) belongs to the *Araliaceae* ginseng family and is a well-known herb that grows only in Canada and the U.S.A. [11]. In 1975, this plant was brought to China. In 1988, American ginseng in China was authorized as a drug by the Ministry of Public Health of China [12]. American ginseng has many functional components, such as sugar, saponin, and volatile oil, among which polysaccharide is an important active component; these molecules have attracted increasing attention by researchers and are indispensable for the therapeutic effects of American ginseng [13–16].

In recent decades, some studies on polysaccharides alleviating antibiotic-associated diarrhoea have been published. For instance, crude sulphated polysaccharides were separated from the red seaweed *Gelidium pacificum* Okamura and then purified to obtain GPOP-1, which changed the structure and composition of the gut microbiota in mice with AAD by improving the richness of useful bacteria, including *Bacteroides*, *Oscillospira*, and *Bifidobacterium*, and reducing the richness of *Parabacteroides* and *Sutterella* [17]. Water-soluble polysaccharides isolated from *Astragalus membranaceus* (WAP) dry roots had advantageous effects on rats with AAD by maintaining the gut structure, regulating the gut microbiota, and increasing SCFA production [18]. The crude polysaccharides obtained from the dry roots of *Pueraria lobata* (PPL) reduced the abundance of beneficial bacteria, including *Oscillospira* and *Anaerotruncus*. This result indicated that PPL intervention could affect rats with AAD by regulating gut microbiota diversity and composition [19]. In previous studies by our group, American ginseng improved the abundance and diversity of gut microbiota by promoting the repair of the intestinal structure in rats with AAD caused by clindamycin phosphate [20]. Most different plant polysaccharides positively regulate the gut microbiota by improving the growth speed of useful bacteria and controlling the proliferation of harmful bacteria. Nevertheless, only a few studies have investigated the influence of American ginseng polysaccharides on antibiotic-related side effects.

The AAD rat model was used in our research, and methods such as conventional HE staining, ELISAs, and 16S rRNA high-throughput sequencing were used to evaluate the influence of American ginseng polysaccharides on rat diarrhoea, intestinal structural damage, levels of inflammatory cytokines, gut microbiota diversity and composition, and SCFA production.

## 2. Materials and Methods

### 2.1. Materials

*P. quinquefolius* was gathered in Fusong, Jilin, China. Lincomycin hydrochloride was purchased from CR Double-Crane Pharmaceuticals Co., Ltd. (Jinan, China), and the TIANamp Stool DNA Kit (DP328) was obtained from Tiangen Biotech Co., Ltd. (Beijing, China). The remaining materials and reactants were purchased from Sinopharm Chemical Reagent Beijing Co., Ltd. Occludin (E-5) (sc-133256), Claudin-1 (A-9) (sc-166338), ERK (D-2) (sc-1647), p-ERK (E-4) (sc-7383), JNK (D-2) (sc-7345), p-JNK (G-7) (sc-6254), p38*α*/*β* MAPK (A-12) (sc-7972), p-p38 MAPK (D-8) (sc-7973), GAPDH (G-9) (sc-365062): Santa Cruz Biotechnology, Inc.; rat tumor necrosis factor alpha (TNF-*α*) (ml001543), rat interleukin 6 (IL-6) (ml001532), rat interleukin 4 (IL-4) (ml001526), rat interleukin 10 (IL-10) (ml001519), rat interleukin 1*β* (IL-1*β*) (ml001554), rat interleukin 17A (IL-17A) (ml027425) kits: Shanghai Enzyme-Linked Biotechnology Co., Ltd. (MLBIO).

### 2.2. Extraction of *P. quinquefolius* Polysaccharides

*P. quinquefolius* polysaccharides (WQP) were obtained as described previously [20]. Specifically, the dried roots of *P. quinquefolius* (500 g) were cut into small pieces of 1 centimeter and suspended in 8 L of distilled water for 2 h and heated at 100°C for 4 h and then filtered in a 120-mesh gauze filter. The resulting filter residue was reextracted twice, and each time 2 h, combined with the resulting extract, concentrated to 1.5 L using a single frying machine. The concentrated solution was centrifuged (4500 rpm, 10 min); the supernatants were taken. Four times the volume of anhydrous ethanol was added to the supernatants to precipitate it, and then, it was left standing for more than 6 h, centrifuged (4500 rpm, 10 min), and the precipitates were taken. The precipitates were dissolved in 800 mL of distilled water and then centrifuged (4500 rpm, 10 min); the supernatants were taken again. Four times the volume of anhydrous ethanol was added to the supernatant to precipitate it, and it was left standing for more than 12 h and then centrifuged (4500 rpm, 10 min); the precipitates were taken. The precipitations were dissolved in 800 mL of distilled water; Sevag reagent (chloroform : n‐butyl alcohol = 4 : 1, *v* : *v*) was used three times to remove the protein layer. The polysaccharide solution layer was collected, and anhydrous ethanol was added to the final concentration of 80% ethanol and then centrifuged (4500 rpm, 10 min); the precipitations were taken and vacuum freeze-dried to yield water-soluble polysaccharides from *P. quinquefolius* (WQP) which were obtained.

### 2.3. Physiochemical Analysis of WQP

All components of carbohydrates, uronic acid, and protein contents were processed as described previously [21–23]. Monosaccharide composition analysis was conducted as described above [24]. Sample preparation: the monosaccharide composition of WQP samples was determined by PMP-HPLC. Then, 2 mg of the polysaccharide sample was taken for complete acid hydrolysis, adding 0.5 mL of 2 M hydrochloric acid methanol solution, filling N_2_ to seal the tube, hydrolysis for 16 h at 80°C, air drying, adding 0.5 mL of 2 M trifluoroacetic acid, hydrolysis for 1 h at 120°C, and then moving into an evaporation dish, 45°C water bath, repeatedly adding anhydrous ethanol to remove trifluoroacetic acid, and drying. Then, 0.5 mL PMP reagent and 0.3 M NaOH solution were added to the hydrolyzed monosaccharide sample, and 0.1 mL water bath was taken for 70°C reaction 30 min after full dissolution for derivatization. After derivatization, 0.05 mL 0.3 M HCl was added, fully mixed, and then extracted with trichloromethane for 3 times. The PMP was removed and transferred to a liquid flask for detection through 0.22 *μ*m microporous membrane.

Standard preparation: 2 mg monosaccharide was used for derivatization, and the treatment method was the same as that of polysaccharide samples.

The chromatographic conditions: Hypersil ODS2 C18 column (4.6 mm × 250 mm, 5 *μ*m). The mobile phase consisted of 0.1 mol/L 82% phosphate buffer solution (pH = 7) and 18% acetonitrile (*v*/*v*). The detection wavelength was 245 nm, injection volume 20 *μ*L, and the flow rate was 1.0 mL/min.

### 2.4. Animal Ethical Statement

Male Wistar rats (180 ± 20 g) were purchased from Changsheng Laboratory Animal Technology Co., Ltd. (Liaoning, China) and housed at 22 ± 0.5°C, 50 ± 5% humidity, and a light and dark cycle for twelve hours : twelve hours. The animals were given unlimited access to a laboratory diet and water. These rats were cared for based on the Guidelines for the Care Use of Laboratory Animals, encouraged by the Chinese Legislation on Laboratory Animals, Chinese Academy of Agricultural Sciences, and the Institute of Special Animal and Plant Science. All efforts were made to maximize the protection of rats, reduce their pain, and reduce the total number of rats in the experiment.

### 2.5. In Vivo Experimental Design

After a 7-day adaptation period, the rats were arbitrarily assigned to 6 groups with 6 rats in each: the control group (C), natural recovery group (NR), antibiotic-associated diarrhoea group (DM), and 3 WQP groups (low-dose, medium-dose, and high-dose groups: L, M, and H). According to the Chinese Pharmacopoeia (2020 edition), the intragastric dose was converted from 6 g/60 kg daily dose of human body, and the medium dose was selected 100 mg/kg/day in rats, while the low dose and high dose were 50 mg/kg/day and 200 mg/kg/day, respectively.

For establishment of the AAD model, the rats in the DM group, the NR group, and the three WQP groups were given lincomycin hydrochloride (750 mg/kg) twice a day for 5 days by gavage, and the C group rats were given the same amount of normal saline. Five days later, the rats in the DM group were narcotized by isoflurane through a small narcotic machine. Faecal samples (>0.5 g) were obtained under aseptic conditions and kept at -80°C. The ileal and colon specimens were stored in 10% neutral formalin. The rats were then euthanatized with carbon dioxide.

After 7 days, the rats from the 3 WQP groups were given different doses of WQP every day (50 mg/kg for L; 100 mg/kg for M; 200 mg/kg for H), while the C group rats and the NR group rats were intragastrically administered an equal amount of normal saline. After recovery, blood, ileal, and colon specimens and faecal samples were obtained as described above.

Based on the reference standards (Table 1) [18], diarrhoea of the rats was assessed daily and recorded, as well as their weight and water intake.

### 2.6. Histological Monitoring of the Ileum and Colon

Ileal and colon samples were fixed in 10% formalin, dehydrated in ethanol, embedded in paraffin, cut into pieces (4-5 *μ*m), and stained with haematoxylin and eosin (H&E). The samples were observed with an Olympus IX53 microscope (Japan).

### 2.7. Determination of Cytokine Levels in the Colon Tissues

The colon tissues of the rats were cut and weighed, 0.45 mL of PBS (pH = 7.4) was added for every 50 mg of tissues, and the tissue specimens were sufficiently homogenized in a homogenizer. The samples were centrifuged (3000 rpm, 20 min) at 4°C. The supernatants were carefully collected and stored in EP tubes. After the preparation, the remaining samples were frozen. An enzyme-linked immunosorbent assay was used, and the complete process was conducted based on the instructions. The bicinchoninic acid assay protein concentration assay kit (enhanced) was used to determine the concentration. The cytokines were prepared for analysis.

### 2.8. Microbiota Analysis

The samples were prepared for the high-throughput sequencing analysis of the entire DNA, and Illumina sequencing was performed as previously reported [25]. The V3–V4 region of the 16S rRNA gene was chosen for PCR amplification by a forward primer (5′-ACTCCTACGGGAGGCAGCA-3′) and a reverse primer (5′-GGACTACHVGGGTWTCTAAT-3′). ASV (amplicon sequence variants)/OTU (operational taxonomic units) were compared by quantitative analysis with the microbial ecology (QIIME2) platform, R software (ver. 3.2.0), and the Greengenes database [26, 27]. Alpha diversity, beta diversity, and different species screening were analysed based on OTUs/ASVs and taxonomic ranks. The total original sequence was stored in the NCBI Sequence Read Archive.

### 2.9. SCFA Analysis

The faecal content of every rat was obtained, and SCFAs were assessed as previously described [25]. The caecal contents of every rat (100 mg) were placed in a centrifuge tube and dissolved in a solution of 10 *μ*L of 15% orthophosphoric acid, 100 *μ*L of adipic acid (50 *μ*g/mL, internal standard), and 400 *μ*L of ether. The mixtures were rotated for 1 min and centrifuged at 4°C (12000 rpm, 10 min), and the supernatant was strained by a 0.45 *μ*m compatible membrane filter of organic specimens in the experiment. Standard solutions of propionic acid, butyric acid, and acetic acid at different concentrations were added in ether. These tests were conducted by an Agilent 6890N/5975BGC-MS system (Agilent, Santa Clara, CA, USA). Compounds were isolated by an Agilent HP-INNOWAX capillary column (30 m × 0.25 mm, 0.25 *μ*m). The initial temperature of the oven was 90°C for 3 min, before increasing to 120°C by 10°C/min, 150°C by 5°C/min, and 250°C by 25°C/min for 2 minutes. A temperature of 230°C was maintained to maintain the ion source, and that of the injection port was 250°C. A microlitre of solution was added. The helium flow rate was 1 mL/min, and the split ratio was 10 : 1. Full scan and SIM scan mode electron bombardment ionization (EI) sources were used for mass spectrometry analysis with an electron energy of 70 eV.

### 2.10. Western Blot Analysis

The 200 *μ*L RIPA lysis buffer was added for every 20 mg of colon tissue. Take about the rat colon tissue was placed in a tissue homogenizer, RIPA lysis buffer with protease as well as phosphatase inhibiting agents was added and the sample was crushed on ice until homogenized, placed on ice for 30 min, and centrifuged (12000 rpm, 10 min). Then, the supernatant was collected. A BCA protein detection kit was used to measure the protein concentration. Forty micrograms of colon histone protein was transferred to a PVDF membrane by SDS-PAGE. The membrane was sealed at room temperature for 2 h using 5% skim milk powder. The primary antibody was stored overnight at 4°C, and the secondary antibody was stored for only 2 h. Protein bands were measured by a U.S. ChemiDoc™ MP Imaging System. ImageJ software was used to quantitatively determine the optical density.

### 2.11. Statistical Analysis

Statistical analysis was carried out using GraphPad Prism 5.0. software. Analysis of variance and Tukey's test were adopted to compare different groups. All data are expressed as the mean ± standard deviation (SD). Significance was defined as *P* < 0.05.

## 3. Results

### 3.1. Physicochemical Properties of WQP

The yield of WQP was 6.71% (*w*/*w*). The carbohydrate, uronic acid, and protein contents were 85.2%, 31.9%, and 2.1%, respectively. The results showed that WQP was primarily composed of glucose (33.2%), galactose (8.9%), arabinose (12.2%), galacturonic acid (43.9%), and a small amount of rhamnose (1.8%).

### 3.2. Normal Status of the Rats

**Figure 1:**
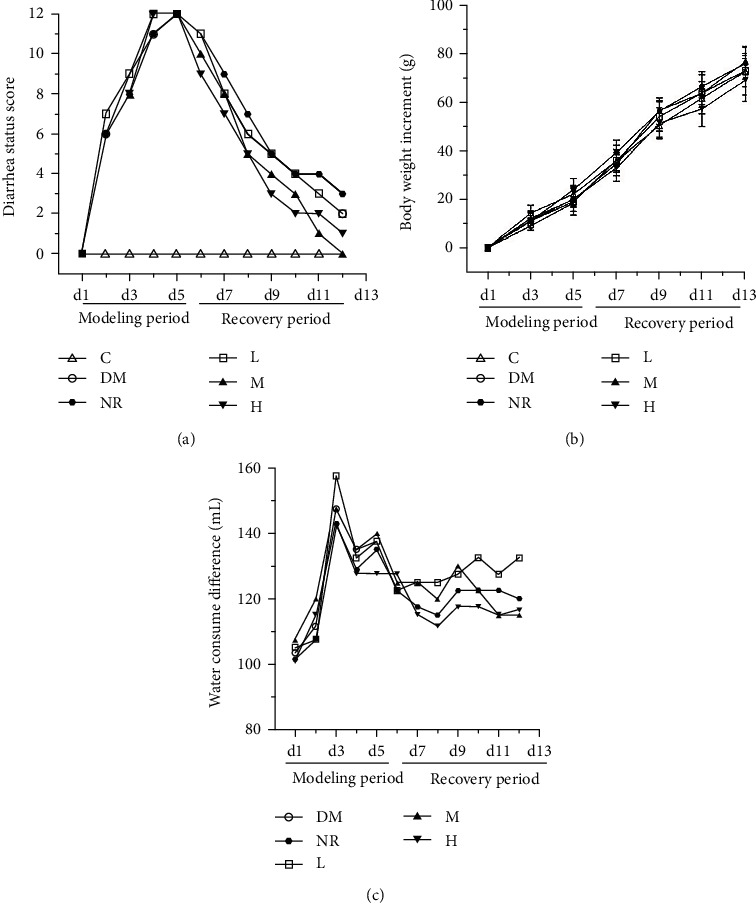
Effects of WQP on (a) total diarrhoea status scores, (b) body weight increment, and (c) the difference in water consumption. C: control group; DM: antibiotic-associated diarrhoea group; NR: natural recovery group; L: low-dosage WQP group; M: medium-dosage WQP group; H: high-dosage WQP group. Data are expressed as means ± SD (*n* = 6).

After 3 days of intragastric administration of lincomycin hydrochloride, the rats began to develop diarrhoea to varying degrees and showed symptoms such as drowsiness, decreased activity, inactivity, increased water intake, and increased defecation times, which were aggravated with the prolongation of administration time. In severe cases, redness and swelling of the anus and faecal adhesion were observed. After modelling, all model animals developed severe diarrhoea, the faeces became watery and soft, and the diarrhoea status scores peaked, confirming that the AAD rat model was successfully established, as shown in Figure 1. From the 6th day, the animals in the NR group were given normal physiological saline via intragastric administration as the control to simulate the natural recovery state, while the animals in the other groups were given intragastric administration of L, M, and H. On the 7th day of administration, the diarrhoea score of group M decreased to zero, and no diarrhoea was observed. A small number of rats in the NR, L, and H groups still had diarrhoea. During the recovery period, the weight recovery of the rats in the NR group was less than that in the WQP group, indicating that these different doses of WQP could significantly reduce the diarrhoea score, promote the weight gain, and improve the general state of the rats.

### 3.3. Histopathological Analysis of the Ileal and Colon Tissues

**Figure 2:**
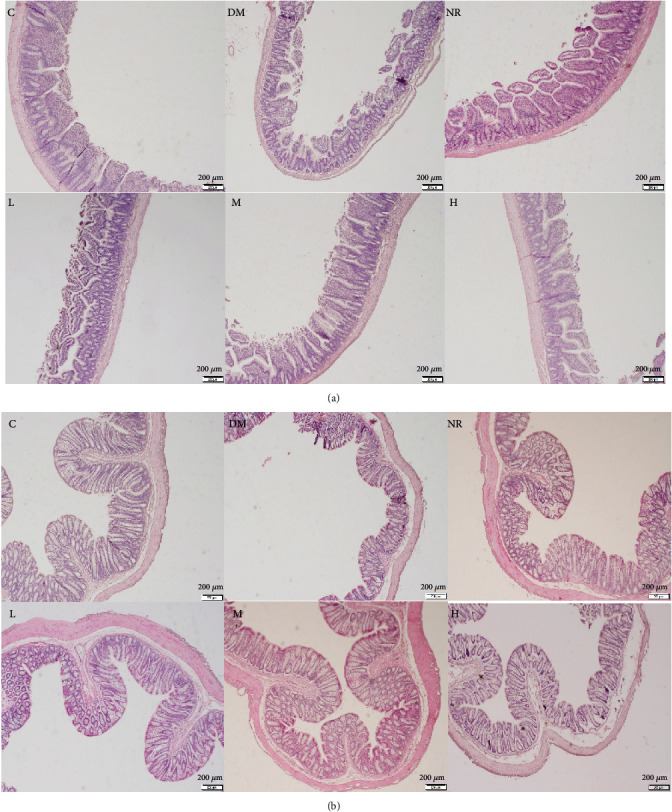
Histopathological observation of the intestine (×40): (a) ileum; (b) colon. C: control group; DM: antibiotic-associated diarrhoea group; NR: natural recovery group; L: low-dosage WQP group; M: medium-dosage WQP group; H: high-dosage WQP group.

Figure 2 illustrates the histopathological variations of the ileum and colon among various groups. As shown in Figure 2(a), the ileal histological morphology of the rats in group C was normal, with slender and neat villi. In the DM group, the villi were shorter, intestinal mucosal epithelial cells were atrophied, and ileal submucosa and villus interstitial oedema occurred. The length of the intestinal villi in the NR group that simulated natural recovery somewhat recovered, but there was obvious inflammatory infiltration. Compared with that in the NR group, the mucosal damage in the groups with different doses of polysaccharides significantly recovered, the villi in group M were slender and neat, and the oedema was weakened; and the ileal tissue damage in groups L and H was also recovered to a certain extent, but the effect was not as obvious as that in group M.

As shown in Figure 2(b), the colon structure of rats in the C group was intact, with orderly arrangement of microvilli on the epithelial surface of the intestinal mucosa and abundant goblet cells. In the DM group, the colonic villi were short and sparse, the epithelial cells were disordered, interstitial oedema was obvious, and the crypts were shallow. The colonic structure of the NR group was better than that of the DM group. The structural recovery of the polysaccharide groups was good compared to that of the NR group. The M group intestinal villi were more slender, orderly, and dense, the crypt was deepened, the interstitial oedema of the villi was weakened, and the number of cup cells was increased. The recovery of the colon tissues of the L and H groups was not as obvious as that of the M group. Therefore, WQP can improve the intestinal tissue damage caused by lincomycin hydrochloride in rats and restore the integrity of intestinal structure to a certain extent.

### 3.4. Changes in Inflammatory Cytokines

**Figure 3:**
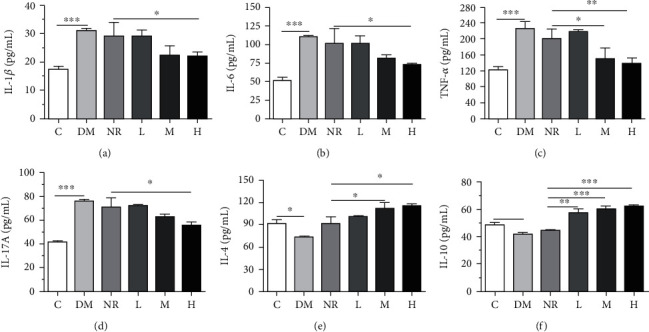
Levels of inflammatory cytokines: (a) Il-1*β*; (b) IL-6; (c) TNF-*α*; (d) IL-17A; (e) IL-4; (f) IL-10. C: control group; DM: antibiotic-associated diarrhoea group; NR: natural recovery group; L: low-dosage WQP group; M: medium-dosage WQP group; H: high-dosage WQP group. Data are expressed as means ± SD (*n* = 6). ^∗^*P* < 0.05, ^∗∗^*P* < 0.01, and ^∗∗∗^*P* < 0.001.

Figures 3(a)–3(d) show that the levels of Il-1*β*, IL-6, TNF-*α*, and IL-17A in the colon tissues of the DM group were greater than those in the C group (*P* < 0.001), which may be due to the increased levels of the proinflammatory factors IL-1*β*, IL-6, TNF-*α*, and IL-17A. Compared with those of the NR group, the levels of IL-1*β*, IL-6, TNF-*α*, and IL-17A in group H were substantially decreased (*P* < 0.05), and the levels of IL-1*β*, IL-6, TNF-*α*, and IL-17A in group M were decreased.

Figures 3(e) and 3(f) show that the IL-4 and IL-10 levels in the colon tissue of the DM group were largely reduced compared to those of the C group (*P* < 0.05), which may be due to the imbalance of gut microbiota leading to decreased levels of the anti-inflammatory factors IL-4 and IL-10. Compared to that of the NR group, the amount of IL-4 and IL-10 in the M/H groups was strongly increased (*P* < 0.05), and the cytokine contents in the L group were also improved, but there was no obvious difference.

### 3.5. Effects of WQP on Gut Microbiota Composition and Diversity

#### 3.5.1. *α*- and *β*-Diversity Analysis of the Gut Microbiota

**Figure 4:**
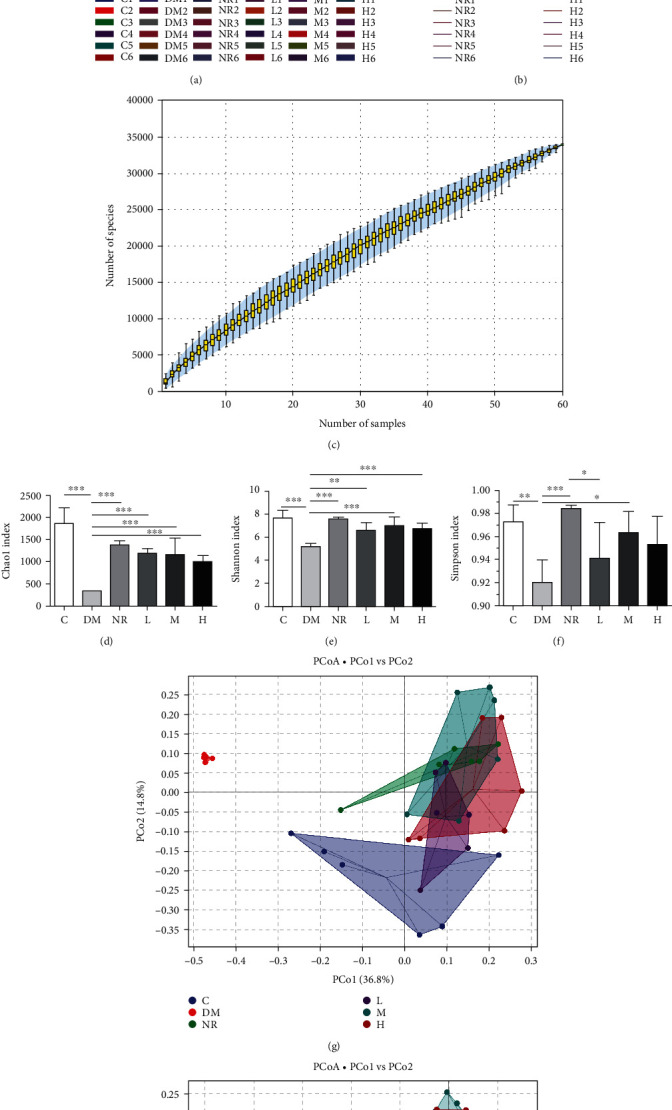
*α*- and *β*-diversity analysis of the gut microbiota. (a) Observed species curve. (b) Rank abundance curve. (c) Specaccum curve. (d) Chao1 index. (e) Shannon index. (f) Simpson index. (g) PCoA based on weighted UniFrac distances. (h) PCoA based on unweighted UniFrac distances. C: control group; DM: antibiotic-associated diarrhoea group; NR: natural recovery group; L: low-dosage WQP group; M: medium-dosage WQP group; H: high-dosage WQP group. Data are expressed as means ± SD (*n* = 6). ^∗^*P* < 0.05, ^∗∗^*P* < 0.01, and ^∗∗∗^*P* < 0.001.

The Illumina MiSeq system was used to study the influences of various WQP doses on the gut microbiota structure of the rats with AAD. The species diversity of the samples was statistically evaluated by the alpha diversity analysis index. A rarefaction curve can be adopted to compare the sample species diversity with various sequencing data volumes to determine if these sequencing statistical volumes of the samples are logical. The rank abundance curve can indicate both the abundance and evenness of species. If the curve is stable, it means that the species are evenly distributed. A specaccum curve was adopted to evaluate and predict whether species abundance would increase with the size of the sample and is commonly used to estimate whether the sample size is sufficient and evaluate community abundance. Rarefaction curves (observed species index curve) were gentle at the end (Figure 4(a)). The curves of rank abundance were stable (Figure 4(b)). As the number of samples increased, the specaccum curve first increased and then remained stable (Figure 4(c)). The results reflected that at the current sequencing depth, the sequencing data were sufficient to indicate the diversity contained in the current samples, each of which could be adopted for the following analysis. The Chao1 index reflects the richness of gut microbiota, and the larger the index is, the greater the community abundance is. The Shannon and Simpson indices reflect gut microbiota diversity, and the greater the value is, the greater the community diversity is. Compared with those of the C group, the richness and diversity were notably decreased in the DM group (*P* < 0.01), as shown in Figures 4(d)–4(f). In contrast to those in the DM group, the richness and diversity of the NR group and the different WQP groups were notably increased (*P* < 0.05), indicating that different WQP groups showed increased gut microbiota abundance as well as diversity.

The gut microbiota *β-*diversity was estimated by PCoA with weighted UniFrac distance and unweighted UniFrac distance to confirm the similarities of the microbial community composition in the corresponding dimensions of samples between groups. As shown in Figures 4(g) and 4(h), PCoA indicated that the points of samples in the DM group and representative samples in the C group were significantly different, demonstrating that the rat gut microbiota structure varied significantly after modelling. Most points representing samples in the NR group and different WQP groups were clustered together, indicating that the microbiota composition and structure were similar. These results suggest that WQP restores the gut microbiota, especially after antibiotic treatment.

#### 3.5.2. Analysis of Gut Microbiota Composition

**Figure 5:**
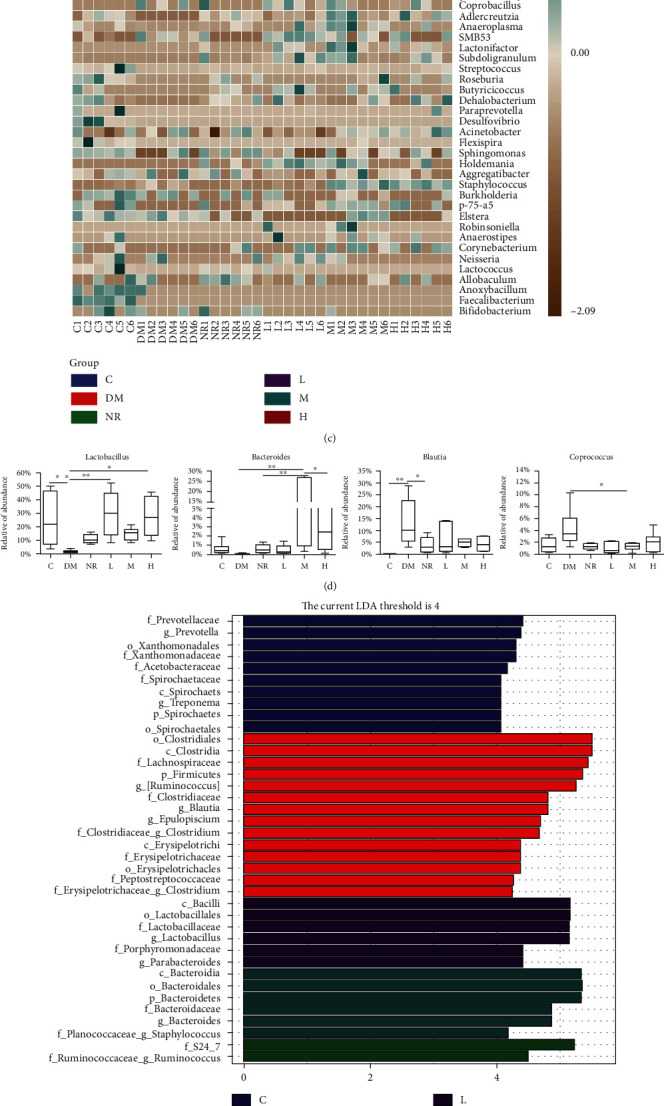
WQP modulated gut microbiota at phylum and genus levels and LEfSe analysis of the dominant biomarker taxa among the six groups. (a) Phylum level. (b) Ratio of Firmicutes to Bacteroidetes. (c) The heat map of taxa in six groups at genus level. (d) Effects of WQP on the level of the representative bacteria at genus level. (e) LDA score distribution histogram. (f) Evolutionary cladogram. The threshold score of LDA was 4.0. C: control group; DM: antibiotic-associated diarrhoea group; NR: natural recovery group; L: low-dosage WQP group; M: medium-dosage WQP group; H: high-dosage WQP group. Data are expressed as means ± SD (*n* = 6). ^∗^*P* < 0.05, ^∗∗^*P* < 0.01, and ^∗∗∗^*P* < 0.001.

After 16S rRNA sequencing, the researchers tested the gut microbiota structure at various levels. The gut microbiota of rats in these groups, at the phylum level, was mainly composed of Firmicutes, Bacteroidetes, Proteobacteria, and Spirochaetes, among which Firmicutes had the greatest abundance (Figure 5(a)). Bacteroidetes was the second most abundant group. Notably, in contrast to that of the C group, the related richness of Firmicutes increased in the DM group, and the relative richness of Bacteroidetes, Proteobacteria, and Spirochetes decreased. At the phylum level, compared with that of the C group, the ratio of Firmicutes to Bacteroidetes in the DM group increased, and it was remarkably decreased after different doses of WQP (*P* < 0.001), as shown in Figure 5(b). The structural differences in the gut microbiota at the genus level are depicted in the heat map (Figure 5(c)). During the long-term experiment, as shown in Figure 5(d), compared with that of the C group, the relative richness of *Lactobacillus* in the DM group substantially decreased (*P* < 0.05), and the relative richness of *Bacteroides* decreased, indicating no obvious difference. In contrast, the relative richness of *Blautia* significantly increased (*P* < 0.01), and the relative abundance of *Coprococcus* increased, also indicating no obvious difference. Moreover, different doses of WQP increased the relative richness of *Lactobacillus* and *Bacteroides* and reduced the relative richness of *Blautia* and *Coprococcus*, affecting the gut microbiota composition. In contrast to that of the DM group, the gut microbiota of the NR group and the different WQP groups showed a certain recovery. These results suggest that WQP can alleviate diarrhoea symptoms by adjusting the gut microbiota composition as well as diversity.

The LEfSe method was used to determine the faecal microbial taxa with the greatest difference in each group. The researchers tested whether each group's gut microbiome was made up of a specific group of bacterial taxa. The LEfSe method is an association of nonparametric tests and linear discriminant analysis, which can test the difference in microbiota abundance. With the LDA score set at 4.0 as the screening standard, the microbes with high abundance in this group can be determined. At the genus level, LEfSe analysis, as shown in Figures 5(e) and 5(f), and the LDA score distribution histogram showed, more intuitively, that the C group had the highest significance for *g_Prevotella*; the DM group had the highest significance for *g_[Ruminoccus]*; the NR group had the highest significance for *f_Ruminococcaceae_g_Ruminococcus*; the L group had the highest significance for *g_Lactobacillus*; the M group had the highest significance for *g_Bacteroides*; and the H group showed no differentially abundant bacteria. In conclusion, these results further indicated that different doses of WQP can alter the gut microbiota structure, enhance the recovery of the microbiota, and improve the composition of the gut microbiota in a rat model of antibiotic-associated diarrhoea.

### 3.6. Analysis of SCFAs

**Figure 6:**
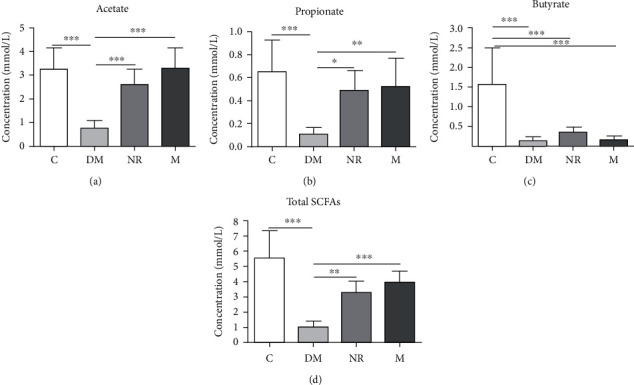
Effects of WQP on SCFA concentrations in feces of rats. C: control group; DM: antibiotic-associated diarrhoea group; NR: natural recovery group; M: medium-dosage WQP group. Data are expressed as means ± SD (*n* = 6). ^∗^*P* < 0.05, ^∗∗^*P* < 0.01, and ^∗∗∗^*P* < 0.001.

The SCFAs in the faeces of rats in each group were mainly composed of acetate, propionate, and butyrate. The acetate contents were the highest, followed by those of propionate and butyrate. The total SCFA content was the sum of the contents of propionate, acetate, isobutyrate, valerate, butyrate, and isovalerate. The contents of acetate, propionate, butyrate, and total SCFAs in the DM group were substantially lower than those in the C group (*P* < 0.001), as shown in Figure 6. In contrast to those in the NR group, the contents of propionate, acetate, and total SCFAs in group M were increased.

### 3.7. Effects of WQP on the Expression of MAPK Pathway Components

**Figure 7:**
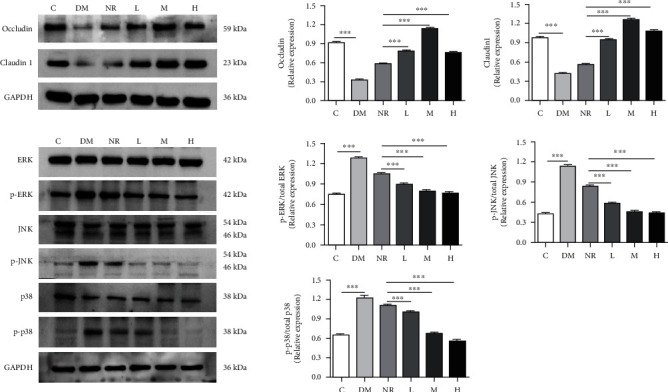
Expression of tight junction proteins in rat colon and WQP blocked the lincomycin hydrochloride-induced activation of MAPK pathways. C: control group; DM: antibiotic-associated diarrhoea group; NR: natural recovery group; L: low-dosage WQP group; M: medium-dosage WQP group; H: high-dosage WQP group. Data are expressed as means ± SD (*n* = 6). ^∗^*P* < 0.05, ^∗∗^*P* < 0.01, and ^∗∗∗^*P* < 0.001.

The expression of the tight junction proteins Occludin and Claudin-1 in the colon tissues of the rats was determined (Figure 7). Compared with that of the C group, the expression of Occludin and Claudin-1 in the DM group substantially decreased (*P* < 0.001). Compared with the NR group, the different WQP groups showed strongly increased expression of Occludin and Claudin-1 (*P* < 0.001). This finding indicated that different doses of WQP could increase the expression levels of tight junction proteins, which were protective in the intestinal barrier. In addition, the expression of ERK, p-ERK, JNK, p-JNK, p38, and p-p38 in the rat colon tissues was determined. Compared with those of the C group, the p-ERK, p-JNK, and p-p38 levels in the DM group were significantly upregulated (*P* < 0.001). Compared with the NR group, the different WQP groups showed strongly decreased expression of p-ERK, p-JNK, and p-p38 (*P* < 0.001). These results suggest that WQP exerts its intestinal protective effects by blocking the inflammatory MAPK signaling pathway.

## 4. Discussion

The gut microbiota is a complex ecosystem and is closely associated with health and disease [28]. Antibiotics are often used to treat infections, inflammation, etc. AAD is a main side reaction during antibiotic treatment and is related to inflammation, gut microbiota, and intestinal structural changes [29, 30]. In this study, the ameliorative effect of *P. quinquefolius* polysaccharides on antibiotic-related side effects was assessed in a rat model of lincomycin hydrochloride-induced AAD. The intestinal tract is the main place for nutrient absorption, and the absorption and utilization of nutrients are determined by the state of the intestinal villi; in general, the length of the intestinal villi is positively correlated with the absorptive capacity [31]. During the study, the rats with AAD showed clinical symptoms of diarrhoea, decreased weight, increased water absorption and inflammatory cytokine levels, and decreased SCFA concentrations, indicating that the rat model of AAD was successfully established [18]. Compared with those of the NR group, the pathological characteristics of the ileum and colon in the different WQP groups were significantly alleviated. The levels of the proinflammatory factors IL-1*β*, IL-6, TNF-*α*, and IL-17A decreased, but those of the anti-inflammatory factors IL-4 and IL-10 increased. The contents of acetates, propionates, and total SCFAs increased. Thus, this treatment regulates the gut microbiota composition and structure and improves intestinal health. Interestingly, we observed different doses of WQP groups have different effects on the *α*-diversity of gut microbiota, which could be attributed that different indexes reflect the diversity from different aspects, such as Chao1 index mainly represents species richness, while Shannon index and Simpson index give consideration to evenness of species on the basis of richness, so they can more truly reflect species diversity, and Shannon index can reflect rare OTU, while Simpson index reflects dominant OTU.

Polysaccharides are usually found in plants and can regulate the gut microbiota in different conditions, thereby playing a key role in many chronic diseases [32, 33]. *Astragalus mongholicus* polysaccharides were shown to alleviate nonalcoholic fatty liver disease through gut microbiota regulation in mice [34]. *Phellinus linteus* polysaccharide extracts could improve insulin resistance, ameliorate intestinal dysbiosis, and help maintain intestinal barrier function in a diabetic rat model by regulating gut microbiota composition [35]. *Ganoderma lucidum* polysaccharides could modulate inflammation, gut microbiota, and gut barrier function in rats with high-fat diet- (HFD-) induced obesity [36]. *Scutellaria baicalensis* Georgi polysaccharides, *Ficus carica* polysaccharides, *G. lucidum* polysaccharides, and purple sweet potato polysaccharides could ameliorate DSS-induced ulcerative colitis in rats by regulating gut microbiota [37–40]. Therefore, many polysaccharides can actively improve diseases by regulating the gut microbiota.

At present, herbal medicine has good efficacy in the treatment of diarrhoea with different symptoms and gut microbiota dysbiosis, and the complicated interaction between Chinese herbal medicine and gut microbiota is the key to TCM treatment [41, 42]. For instance, Shenlingbaizhu powder improved the gut microbiota composition and diversity in rats with AAD [2]. Gegen Qinlian decoction (GQT) is a medicinal herb decoction used in China and is commonly adopted to cure gastrointestinal disorders, especially diarrhoea, in the clinic [43]. GQT improves immunity and maintains intestinal barrier functions in people with colorectal cancer through the gut microbiota, specifically through improving the relative abundance of *Bacteroides*, *Akkermansia*, and *Prevotella* and reducing the related richness of Megamonas and Veillonella [44]. Using a novel monofloral honey from a plant used in traditional Chinese medicine, Prunella Vulgaris (PVH), Wang et al. explored that the gastrointestinal protective potential of PVH against dextran sulfate sodium- (DSS-) induced acute colitis in rats and found that PVH exerts anticolitis effects, which may be associated with its regulating effects on gut microbiota [45]. It was reported that polyphenols in propolis can play a protective role against dextran sulfate sodium- (DSS-) induced colitis in rats, by modulating the richness and diversity of gut microbiota [46]. Fermented ginseng with probiotics (*Lactobacillus fermentum*) could relieve AAD symptoms and colon inflammation, decrease the expression of immune elements such as TLR4 and NF-*κ*B in the colon, and restore the gut microbiota to its original state [47]. Our research group previously studied the role of *Panax ginseng* polysaccharides (WGP) on gut microbiota disorder induced by lincomycin hydrochloride. Compared to AAD mice, WGP increased the relative abundance of *Lactobacillus*, *Lactococcus*, and *Streptococcus*, but decreased the relative abundance of *Bacteroides*, indicating that the WGP had a certain recovery effects on gut microbiota [48]. In this study, compared to the AAD rats, different doses of WQP could improve the relative richness of *Lactobacillus* as well as *Bacteroides* and decrease the relative richness of *Blautia* and *Coprococcus*. However, WQP was not found to have the same effect on the abundance of these gut microbiota compared with WGP. This may be because the two animal models are different, which possesses different influences on the gut microbiota, and also indirectly affect the effect of WQP on the dysbiosis of gut microbiota. Some Chinese herbal polysaccharides have positive effects on antibiotic-related diarrhoea, while having different effects on gut microbiota. *Astragalus membranaceus* polysaccharides increased the relative abundance of *Pseudomonas* and decreased the relative abundance of *Allobaculum* and *Coprococcus* in antibiotic-associated diarrhoea rats [18]. *P. lobata* is a popular medicinal and edible herb in China, and *P. lobata* polysaccharides could relieve antibiotic-associated diarrhoea in mice with colonic pathological variations and gut microbiota dysbiosis by increasing the abundances of *Oscillospira* and *Anaerotruncus* [19]. These results also suggest that different sources of polysaccharides showed various effects on gut microbiota, which may be related to the differences of monosaccharide composition, molecular weight, length of the main chain, space conformation, and so on. Although the mechanisms by which WQP or other polysaccharides from plant resources balance the gut microbiota need more research, our results may provide some theoretical basis for similar research. On the one hand, gut microbiota can degrade polysaccharides and promote the body's absorption and utilization of polysaccharides. On the other hand, polysaccharides can regulate the composition of gut microbiota by increasing beneficial bacteria and reducing harmful bacteria, thus improving the body's health level. Herbal medicine can act on AAD, but the specific mechanism and composition of the affected microbiota are not the same; this is consistent with the multitarget activity of herbal medicine.

SCFAs refer to fatty acids with less than 6 carbon atoms, mainly acetate, propionate, and butyrate. SCFAs are the main bacterial metabolites, which are produced by specific colonic anaerobic bacteria after fermentation of nondegradable dietary fiber. *Lactobacillus* produces lactate, *Bacteroides* produces acetate and butyrate, and *Coprococcus* produces butyrate after the fermentation of gut microbiota. Acetate and propionate mainly participate in energy metabolism of the body, while butyrate mainly provides energy for intestinal mucosa. SCFAs have many important biological functions, such as can provide energy for intestinal epithelial cells, reduce intestinal inflammation, regulate gut microbiota, and participate in body immunity. It has been reported that *Lactobacillus* served as carbohydrate-degrading bacteria promoting the decomposition of polysaccharides and is positively correlated with the generation of acetate, propionate, and butyrate, which benefits the gut microecology [49]. It also has been reported that *Bacteroides*, *Blautia*, *Coprococcus*, and so on are all capable of producing short-chain fatty acid bacteria by fermentation in the human intestinal tract, which play a vital important role in maintaining human health [50]. In this study, different doses of WQP by increasing the abundance of *Lactobacillus* and *Bacteroides* are associated with the increase of short-chain fatty acid production. So the improving effects of WQP on the AAD might be correlated with the ability of adjusting the SCFA level through the increasing relative abundance of *Lactobacillus* and *Bacteroides*.

The tight junctions between cells are mainly composed of tight junction proteins, including Occludin and Claudin family proteins [51]. Claudin is a major component of the tight junction complex that alters epithelial permeability. Occludin, a complete membrane protein primarily involved in the tight junctions between epithelial and endothelial cells, is critical for the composition and adjustment of the permeability of the tight junction paracellular barrier. This protein is expressed in cells without tight junctions and induces adhesion. Disruption of the intestinal closure junction barrier, which leads to disruption and inflammation of the mucosal immunity, acts as a development trigger of intestinal as well as systemic disease [52]. In this study, WQP enhanced the expression levels of the tight junction proteins Occludin and Claudin-1, which play a protective role in the intestinal barrier. Similarly, *G. lucidum* polysaccharides were shown to have beneficial effects on gut barrier functions by improving the protein expression of the tight junction proteins Occludin and Claudin-1 [39]. The mitogen-activated protein kinase family (MAPK) is a conserved serine protein kinase family in the cytoplasm that can transmit stress signals and promote cell proliferation and can mediate the signal transduction of various cytokines [53]. The major elements in the MAPK family are ERK, JNK, and p38, connecting various cell surface proteins and the expression of inflammatory genes [54]. The MAPK pathway provides cells with extracellular signals and regulates the inflammatory response [55]. Studies have shown that many physiological and pathological reactions are mediated by the MAPK signaling pathway. *Radix Codonopsis* polysaccharides [56] and *Ganoderma atrum* polysaccharides [57] have been proved to inhibit the production of inflammatory factors by inhibiting the activation of the MAPK pathway, thus avoiding pathological changes caused by excessive release of inflammatory factors. *Cyclocarya paliurus polysaccharide* (CP) alleviates liver inflammation in mice via beneficial regulation of gut microbiota and TLR4/MAPK signaling pathways, suggesting that CP act as prebiotics in ameliorating the liver inflammation in mice through regulating the gut microbiota composition and increasing the concentration of SCFAs [58]. The protective effect of deer oil on alcohol-induced gastric mucosal injury has also been explored by blocking the MAPK signaling pathway [59]. In this study, compared with those of the DM group, the p-ERK, p-JNK, and p-p38 levels in the different WQP groups were substantially lower, showing that WQP is protective in the intestinal tract by blocking the MAPK inflammatory signaling pathway.

## 5. Conclusions

Overall, WQP was primarily comprised of galacturonic acid, glucose, galactose, arabinose, and rhamnose. This treatment can improve the symptoms of diarrhoea, destruction of the intestinal structure, and bacterial imbalance caused by lincomycin hydrochloride by promoting the repair of the intestinal structure of the rats with AAD, regulating inflammatory cytokine levels, increasing SCFA production, and regulating gut microbiota composition and diversity. WQP improved the expression of Occludin and Claudin-1 to protect the intestinal barrier. In addition, WQP inhibited the MAPK inflammatory signaling pathway in the colon tissues. These results indicate that WQP can act as a potential natural product to moderate AAD, and the effects are related to the modulatory effect of WQP on the gut microbiota.

## Figures and Tables

**Table 1:** Diarrhoea status assessment.

Scores	Diarrhoea status
0	Normal
1	Loose, light coloured, and nonsticking perianal stool, normal mental state
2	Adhesive stool in the anus, mental depression, no appetite for food, weight loss

## Data Availability

The data that supports the findings of this study are available in the article.
